# Age-related effects of X-ray irradiation on mouse hippocampus

**DOI:** 10.18632/oncotarget.8575

**Published:** 2016-04-04

**Authors:** Arianna Casciati, Katalin Dobos, Francesca Antonelli, Anett Benedek, Stefan J. Kempf, Montserrat Bellés, Andrea Balogh, Mirella Tanori, Luis Heredia, Michael J. Atkinson, Christine von Toerne, Omid Azimzadeh, Anna Saran, Geza Sáfrány, Mohammed A. Benotmane, M. Victoria Linares-Vidal, Soile Tapio, Katalin Lumniczky, Simonetta Pazzaglia

**Affiliations:** ^1^ Laboratory of Biomedical Technologies, Agenzia Nazionale per le Nuove Tecnologie, l'Energia e lo Sviluppo Economico Sostenibile (ENEA), Rome, Italy; ^2^ National Public Health Center - National Research Directorate for Radiobiology and Radiohygiene, Budapest, Hungary; ^3^ Physiology Unit, School of Medicine, IISPV, Rovira I Virgili University (URV), Reus, Spain; ^4^ Institute of Radiation Biology, Helmholtz Zentrum Munchen, German Research Center for Environmental Health GmbH, Neuherberg, Germany; ^5^ Institute of Radiation Biology, Technical University Munich, Munich, Germany; ^6^ Research Unit Protein Science, Helmholtz Zentrum München, German Research Center for Environmental Health GmbH, Neuherberg, Germany; ^7^ Radiobiology Unit, Belgian Nuclear Research Centre, SCK•CEN, Belgium; ^8^ Present address: Department of Biochemistry and Molecular Biology, University of Southern Denmark, Odense, Denmark

**Keywords:** radiation, hippocampal neurogenesis, mitochondria, proteomics, cognitive effects

## Abstract

Therapeutic irradiation of pediatric and adult patients can profoundly affect adult neurogenesis, and cognitive impairment manifests as a deficit in hippocampal-dependent functions. Age plays a major role in susceptibility to radiation, and younger children are at higher risk of cognitive decay when compared to adults. Cranial irradiation affects hippocampal neurogenesis by induction of DNA damage in neural progenitors, through the disruption of the neurogenic microenvironment, and defective integration of newborn neurons into the neuronal network. Our goal here was to assess cellular and molecular alterations induced by cranial X-ray exposure to low/moderate doses (0.1 and 2 Gy) in the hippocampus of mice irradiated at the postnatal ages of day 10 or week 10, as well as the dependency of these phenomena on age at irradiation. To this aim, changes in the cellular composition of the dentate gyrus, mitochondrial functionality, proteomic profile in the hippocampus, as well as cognitive performance were evaluated by a multidisciplinary approach. Our results suggest the induction of specific alterations in hippocampal neurogenesis, microvascular density and mitochondrial functions, depending on age at irradiation. A better understanding of how irradiation impairs hippocampal neurogenesis at low and moderate doses is crucial to minimize adverse effects of therapeutic irradiation, contributing also to radiation safety regulations.

## INTRODUCTION

The brain is exposed to ionizing radiation in a number of clinical situations, most notably during radiation therapy used for treatment of malignant central nervous system tumors. Especially in children, cranial radiation therapy has been associated with the highest risk of long-term cognitive morbidity [1–7]. With the improvement of technology and limited fraction size, overt tissue damage can be avoided. However, deficits in the neurocognitive functions, including verbal and short-term memory recall, learning, memory, spatial processing, and severe dementia persist [8–10]. Although some deficits can be observed within the first few months following radiation therapy, most occur several months to years later [11]. The mechanisms underlying radiation-induced deficits are still not fully characterized but involve damage in vascular and glial compartments, altered neuronal function, and neuro-inflammatory processes [12]. Moreover, the dentate gyrus (DG) of the hippocampus, one of the main brain regions where neurogenesis takes place throughout life [13], has been shown to be particularly susceptible to radiation [14]. Hippocampal neural stem-cell injury during whole-brain radiotherapy is central to memory decline, and conformal avoidance of the hippocampus is associated with preservation of memory [15].

Age at exposure plays a pivotal role in susceptibility to radiation. The severity of cognitive impairment after cranial irradiation is more pronounced in children younger than 3 years at the time of treatment [16]. Studies on rodents showed a correlation between age-dependent sensitivity of the developing brain to irradiation and number and vulnerability of progenitor cells [17–19]. Age-related differences in the proliferative properties of neural precursor cells, prevalent in the young brain, and in neuro-inflammatory processes, prevalent in the adult brain, have been involved in the differential radiation response of young and adult brain [20–23].

The metabolic status regulates adult hippocampal neurogenesis [24]. Hence, mitochondria-dependent metabolism itself may provide substantial input into the regulatory networks controlling different stages of neurogenesis in the dentate gyrus (DG). The identification of mitochondrial function as an important player in neurogenesis, together with observations that mitochondria are targets for ionizing radiation effects, potentially implied mitochondrial dysfunction in radiation-induced deficit of hippocampal neurogenesis-dependent cognition [25–30].

While knowledge of the effects of low radiation doses is sparse and existing data are mostly based on moderate to high doses (≥ 2 Gy), epidemiological observations raise concern on possible cognitive effects of low doses during the extensive remodeling ongoing in the immature brain [31]. This study was designed to investigate the influence of dose and age at exposure on radiation-induced response in the hippocampus. To this aim, C57BL/6 mice were cranially irradiated at 10 days (10D), i.e., the peak of the brain growth spurt and an extensive remodeling stage of the immature brain - recognized as a critical sensitive period for toxic insults, lasting for the first four weeks of mouse life [32, 33] - or at 10 weeks (10W) of age, after conclusion of this susceptible stage. A single low dose of 0.1 Gy, equivalent to the dose delivered to the brain of an infant during computed tomography of the skull [34], or a moderate dose of 2 Gy, a typical dose fraction used classically in clinical practice, were employed. To clarify the global stress response after irradiation, hippocampal neurogenesis, mitochondrial functions, as well as changes in protein expression have been evaluated at both doses and compared in mice irradiated at different ages. Although most alterations and defects were detected at the higher dose, some of the changes were also evident at low dose. Age at exposure influenced radiation effects, which were mostly exacerbated when irradiation occurred at younger age.

## RESULTS

### Age-dependence of hippocampal neurogenesis

**Figure 1:**
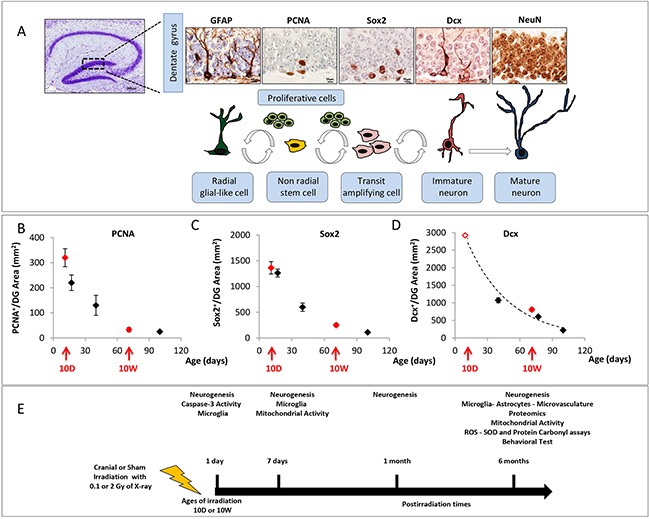
Cell type in neuronal development of the DG and schematic experimental procedure **A.** Immunohistological markers for staging hippocampal neurogenesis. **B.** Age-dependent expression of PCNA, **C.** Sox2 and **D.** Dcx in the DG. Red arrows mark irradiation time (10D at 10W) and red solid symbols indicate the correspondent expression level for each marker; the value of the empty red symbol for Dcx has been extrapolated. DG image 4x magnification, scale bar = 200 μm; images of other markers: 100x magnifications, scale bar = 10 μm. **E.** 10D or 10W old mice were cranially irradiated with 0.1 or 2 Gy of X-rays and analysed at different time points. Bars indicate the times of tissue collection from sham or irradiated mouse groups. (1 and 7 days, 1 and 6 months postirradiation). The number of animals used for molecular analysis is n = 4-5, for behavioral tests is n = 20.

Cellular composition and kinetics of the subgranular and granular zone of the DG can be evaluated through morphological criteria and immunohistochemical analysis of stage-specific neurogenesis markers (Figure 1A). Postnatal hippocampal neurogenesis is a multistep process that recapitulates all stages of neuronal development in a mature central nervous system (CNS). From the largely quiescent neural stem cells (NSCs), also called radial glial-like cells (RGLs), to fully integrated and functional neurons, NSCs and their newborn progeny pass through several developmental stages (Figure 1A). First the quiescent population of NSCs (GFAP^+^) is activated to generate proliferating transit-amplifying cells that enlarge the pool of neurogenic cells called type-2 cells (Sox2^+^). Within the DG, immature neurons migrate up into the granule cell layer, and mature into newborn granule neurons (Dcx^+^) and finally into mature neurons (NeuN^+^) integrating in an existing neuronal circuit. As a first step we characterized hippocampal neurogenesis as a function of age in the DG of unexposed mice. We observed a rapid decline in the expression of several neurogenic markers with progressing age, indicating a drastic decrease in the levels of basal neurogenesis at 10W compared to 10D (−90% PCNA^+^, -82% Sox2^+^, −71% Dcx^+^; Figure 1B–1D).

### Age- and dose-dependent effects of irradiation on hippocampal neurogenesis

Next, we monitored the kinetics of radiation-induced changes in the cellular composition of the DG of 10D- and 10W-irradiated mice by performing the analyses at several time ranging from early (1 and 7 days) to late (1 and 6 months) post-irradiation times (Figure 1E).

**Figure 2:**
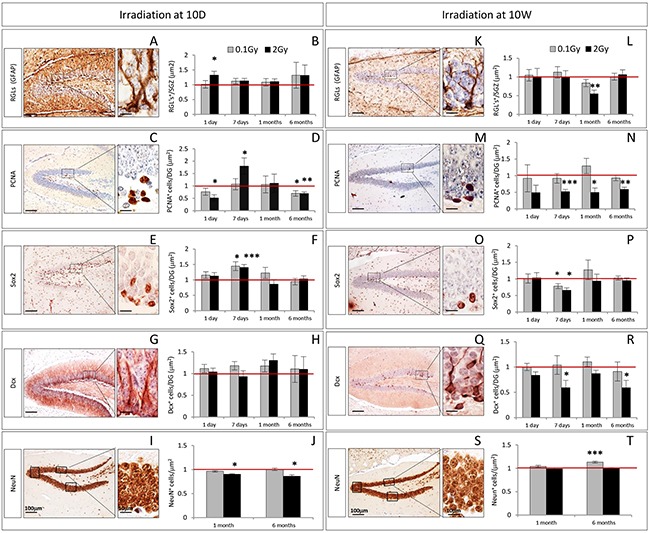
Kinetics of radiation-induced alterations in the cellular composition of the DG after irradiation at 10D and 10W **A–B.** and **K–L.** Immunostaining and quantification for GFAP, **C–D.** and **H–N.** PCNA, **E–F.** and **O–P.** Sox2 and **G–H.** and **Q–R.** Dcx at 1 and 7 days, and at 1 and 6 months after irradiation at 10D and 10W. **I–J.** and **S–T.** Immunostaining and quantification for NeuN at 1 and 6 months after irradiation at 10D and 10W. Images, 10x magnification, scale bar = 100 μm; high magnification 100x scale bar = 10 μm. Data are reported as mean ± SEM *P < 0.05; **P < 0.01; ***P < 0.0001 for comparison with controls (Student-t test unpaired). The number of animals used is n = 4–5.

The earliest alterations in 10D mice irradiated with 2 Gy consisted in an increased expression of GFAP labeling RGL precursors (Figure 2A, 2B, *P* = 0.02) and decreased number of PCNA^+^ cycling progenitors (Figure 2C, 2D, *P* = 0.017), detected at 1 day post-irradiation. One week later, a compensatory increase in the number of PCNA^+^ cells was observed (Figure 2D, *P* = 0.011), accompanied by an increase in the number of Sox2^+^ cells (Figure 2E, 2F, *P* = 0.0007). At 1 month, the only significant perturbation was a depletion in the density of mature granule neurons labelled by NeuN (Figure 2I, 2J, *P* = 0.03). At 6 months post-irradiation, the depletion of NeuN^+^ neurons was still persisting (Figure 2J, *P* = 0.02), along with a decrease in the number of PCNA^+^ cells (Figure 2D, *P* = 0.009). Irradiation of 10D mice at low dose (0.1 Gy) caused a more limited number of changes involving an increased number of Sox2^+^ cells (Figure 2F, *P* = 0.01) 1 week after irradiation, and a decrease in the number of PCNA^+^ cycling progenitors at 6 months (Figure 2D, *P* = 0.04).

In mice of 10W irradiated with 2 Gy, no significant alterations were observed 1 day after irradiation (Figure 2K–2R). Instead, after 7 days significant impairment of the DG subpopulations labelled by PCNA (Figure 2N, *P* = 0.0005), Sox2 (Figure 2P, *P* = 0.01) and Dcx (Figure 2R, *P* = 0.02) were observed. One month post-irradiation, the compartment of PCNA^+^ cycling progenitors was still impaired (Figure 2N, *P* = 0.013) and a significant decrease of RGL labeled by GFAP (Figure 2L, *P* = 0.006) was also observed. The population labeled by PCNA never recovered and number of PCNA^+^ cells remained still depleted at 6 months (Figure 2N, *P* = 0.001), suggesting a permanent effect of radiation injury. At 6 months after irradiation, we also observed a depletion of newborn neurons labeled by Dcx (Figure 2R, *P* = 0.034). Irradiation of 10W-old mice at low dose (0.1 Gy), only caused decrease in the number of Sox2 labelled cells 1 week after irradiation (Figure 2P, *P* = 0.04) and an increased density of NeuN^+^ mature neurons at 6 months (Figure 2T, *P* = 0.0001).

### Apoptosis and inflammatory responses in the adult hippocampus after irradiation at 10D or 10W

**Figure 3:**
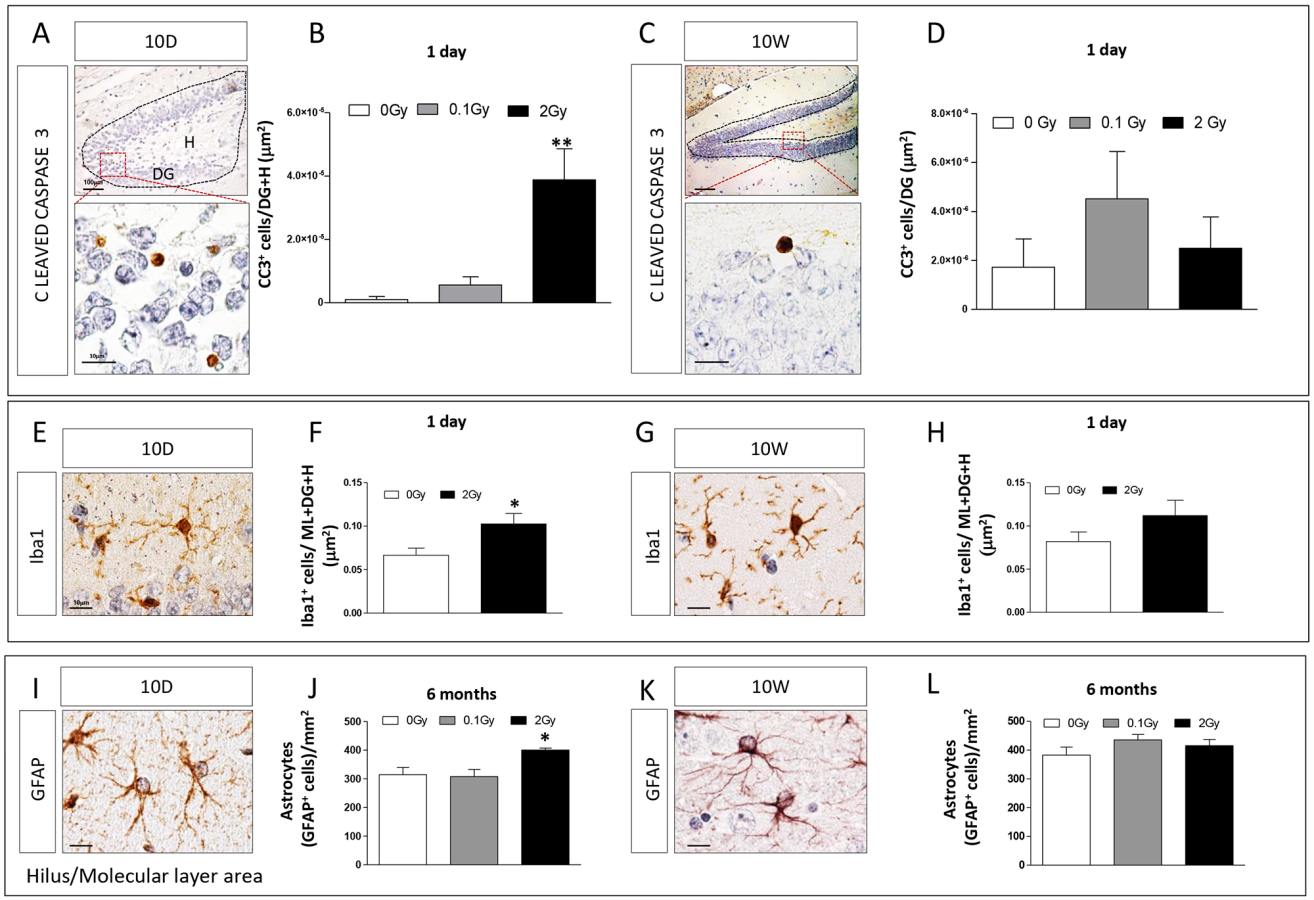
Apoptotic and inflammatory responses in the hippocampus after irradiation at 10D or 10W **A.** and **C.** Expression and **B.** and **D.** quantification of cleaved caspase 3 immunostaining in specific regions (dashed lines) showing a significant increase in apoptosis after 10D- (B), but not 10W-irradiation with 2 Gy (D). Images, 10x magnification, scale bar = 100 μm; high magnification 100x, scale bar = 10 μm. **E.** and **G.** Representative immunostaining for Iba1 in 10D- and 10W-irradiated mice. Quantification of the expression level of Iba1 at 1 day **F–H.** after irradiation with 2 Gy of X-rays at 10D or 10W, showing a significant increase in Iba1 expression only in 10D-irradiated mice at 24 h after exposure. **I.** and **K.** Representative images of GFAP immunostaining at 6 months postirradiation. **J.** Significant increase in the number of GFAP^+^ astrocytes in non neurogenic areas (H and ML) of the hippocampus in 10D-irradiated mice. **L.** No changes were detected in 10W-irradiated mice. Image magnification 100x, scale bar =10 μm. Data are reported as mean ± SEM *P < 0.05; **P < 0.01; comparison with controls. The number of animals used is n = 4–5.

We assessed the presence of apoptotic cells by immunohistochemistry (IHC) against cleaved caspase-3 in the hippocampus of 10D- hilus (H) and DG, (Figure 3A, 3B) and 10W-irradiated mice (DG, Figure 3C, 3D) 1 day after irradiation. Caspase activity was only detected in mice irradiated with 2 Gy at 10D (Figure 3B; 0 Gy *vs* 2 Gy *P* = 0.008), but not in 10W irradiated mice (Figure 3D). Although, after irradiation at 10D, apoptotic cells were also found in other neonatal brain structures, such as the external granule layer of cerebellum (data not shown), the brain weight was not decreased following irradiation, overall suggesting mild brain effects of irradiation at 2 Gy (Supplementary Figure S1).

To investigate the inflammatory response, sections of 10D- and 10W-irradiated mouse brains were immunostained for Iba1, a microglial marker (Figure 3E, 3G). Quantification of the number of Iba1^+^ cells in the whole hippocampus, including DG, molecular layer (ML) and H showed a significant increase in Iba1 expression in 2 Gy-10D-irradiation (Figure 3F, *P* = 0.04) but not in 10W-irradiated mice at 1 day postirradiation (Figure 3H). No increase of Iba1 expression was detected both in 10D- and 10W-irradiated mice 1 and 6 months postirradiation (data not shown). The temporal overlap between increased Iba1 and caspase-3 expression in 10D-irradiated brains (Figure 3B and 3F) is suggestive of a phagocytic function of microglia to remove dead/damaged cells.

Finally, to further investigate long lasting inflammatory consequences of irradiation, we quantified the number of astroglial cells (labeled by GFAP, Figure 3I, 3K) in the H and ML of 10D- and 10W-irradiated mice at 6 months post-irradiation (Figure 3J, 3L). Compared to control mice, we found a significant increase in the number of GFAP^+^ astrocytes at 2 Gy in 10D-irradiated mice (Figure 3J, *P* = 0.017). Instead, no differences were detected in 10D-irradiated mice at a lower dose (0.1 Gy) and in 10W irradiated mice at both doses (Figure 3J, 3L).

### Proteomic analysis in the hippocampus of 10D- and 10W-irradiated mice

**Figure 4:**
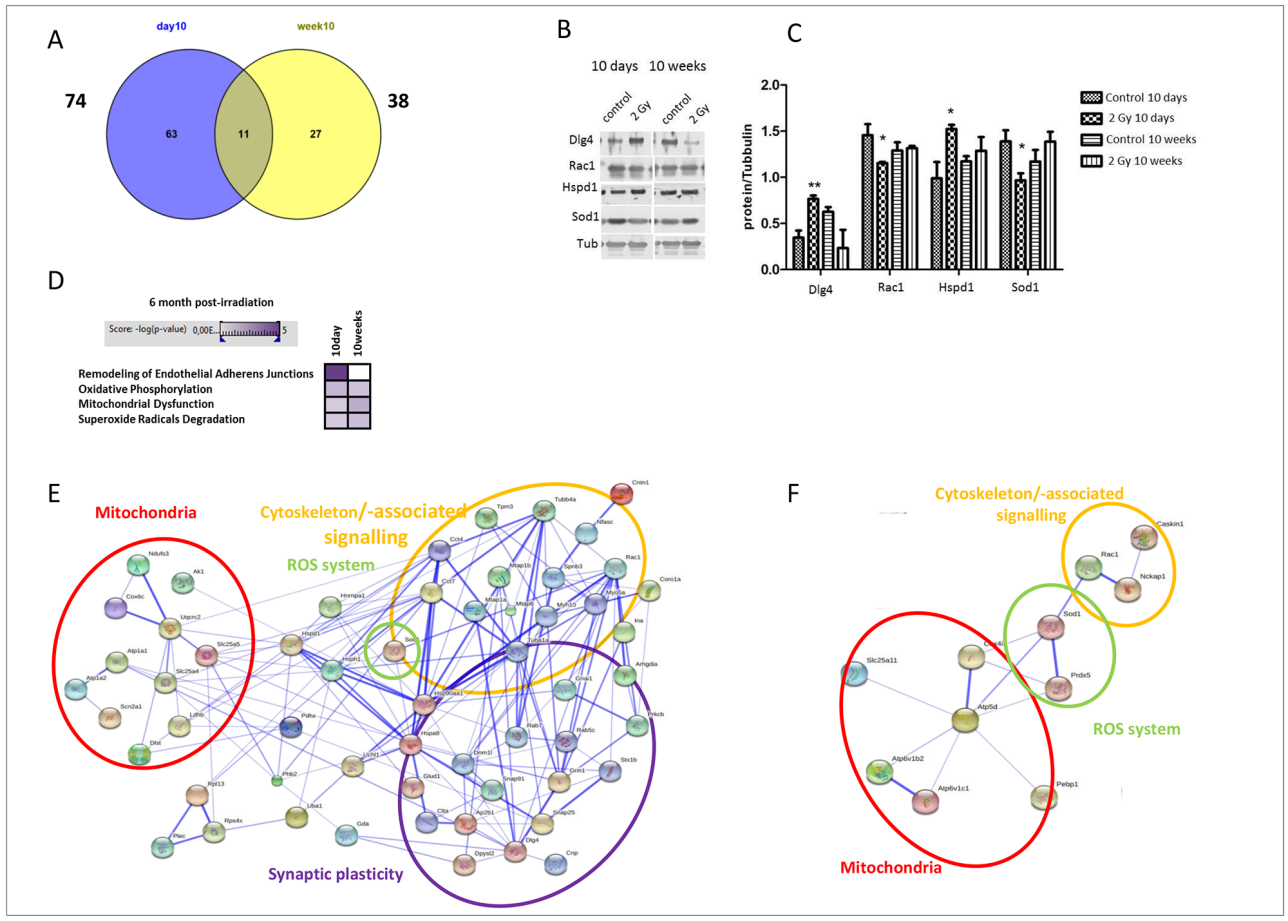
Proteomic analysis and immunoblotting validation of 10D and 10W irradiated mice at 6 months post-irradiation **A.** Venn diagram of all deregulated and shared proteins from global mass spectrometry-based protein quantification in hippocampus of mice irradiated with 2 Gy of X-ray at 10D and 10W. The number outside each circle shows the total number of deregulated proteins. **B.** Immunoblot analysis of postsynaptic protein PSD-95 (Dlg4), Rac1, Hspd1, and Sod1 in protein extracted from 10D- and 10W-irradiated hippocampus. **C.** Graphic representation of densitometric immunoblot analysis in control and irradiated samples. Normalization was performed against alpha-tubulin (Tub) as a loading control (n = 3, unpaired Student's t test, *P < 0.05, **P < 0.01). **D.** Signaling pathway analysis using Ingenuity Pathway Analysis software. High color intensity represents high significance (*P*-value). All colored boxes have a *P* value of ≤0.05; white boxes have a *P* value of ≥0.05. **E.** Protein-protein interaction network of the 10D and **F.** 10W exposed mice analyzed with the STRING software. The number of animals used for proteomic analysis is n = 4.

We quantified the global protein changes in the hippocampus at 6 months after irradiation of 10D- or 10W-old mice with 2 Gy of X-rays using tandem mass spectrometry. The number of significantly deregulated proteins in the hippocampus was 74 and 38 in 10D and 10W irradiated mice, respectively (Figure 4A). Supplementary Tables S1 (10D) and Supplementary Table S2 (10W) show the complete list of identified deregulated proteins. Only a subset of 11 proteins was shared between 10D- and 10W-irradiated mice (Cct4, Caskin1, Hspd1, Tmod2, Prrt2, Uba1, Nptn, SOD1, Pdxp, Rab7 and Rac1), 3 of which showed deregulation of the same direction (Hspd1, upregulated; Pdxp and Rac1, downregulated; Supplementary Table S1 and Supplementary Table S2), suggesting distinct patterns in proteome changes in 10D- and 10W-irradiated mice. To verify the protein expression alterations obtained by the quantitative proteomics, 4 proteins that showed either similar or different pattern of alteration in 10D- or 10W-irradiated mice were chosen for validation by immunoblotting using specific antibodies and tubulin as a loading control. The analysis confirmed a significant increase of postsynaptic protein PSD-95 (Dlg4) and heat shock protein (Hspd1) in 10D-irradiated mice, compared to unexposed controls (Figure 4B, 4C). In good agreement with the proteomics data, immunoblotting analysis also showed decreased expression level of Rac1 and SOD1 in 10D-irradiated mice compared to controls. A slight, not significant, increase of Hspd1 and SOD1 expression level was detected in 10W-irradiated mice.

Bioinformatics analysis of all deregulated proteins was performed using IPA software. In spite of the relatively small number of shared deregulated proteins between the two irradiation time points, the pathways altered by ionizing radiation showed a considerable overlap. The signaling pathways showing significant alterations common to 10D and 10W irradiated mice were “oxidative phosphorylation”, “mitochondrial dysfunction” and “superoxide radicals degradation” (Figure 4D). Instead, the signaling pathway “remodeling of endothelial adherens junctions” was significantly changed only in mice irradiated at 10D (Figure 4D).

Based on Gene Ontology (GO) analysis, proteins associated with subunits of mitochondrial respiratory Complex I (downregulation of Ndufs3), III (downregulation of Uqcrc2), IV (downregulation of Cox6c) and V (upregulation of Atp8a1, Atp1a1 and Atp1a2), as well as antioxidative stress response (downregulation of SOD1) were altered in 10D-irradiated mice (Supplementary Table S1). In 10W-exposed mice, alterations in mitochondrial respiratory Complex IV (decrease in Cox4i1) and V (increase in Atp5d, Atp6v1b2, Atp2b1; decrease in Atp6v1c1) and redox system (increase in SOD1, Prdx5; decrease in Gstp1, and Tmx2) were found (Supplementary Table S2). Proteins associated with mitochondria, antioxidative stress response, and cytoskeleton/-associated signalling formed an interactive network in mice irradiated at 10D (Figure 4E) and 10W (Figure 4F). Noteworthy, the interactive network in 10D-irradiated mice included proteins associated to synaptic plasticity (Figure 4E). The proteomic data built up a more dense network at 10D- compared to 10W-irradiated mice (Figure 4E, 4F). Among specific protein alterations for 10D-irradiated mice, we detected reduced levels of pre-synaptic (Synaptosomal-associated protein of 25 kDa, SNAP 25) and increased post-synaptic proteins [postsynaptic density protein 95, PSD 95 (Dlg4)] (Supplementary Table S1).

### Induction of mitochondrial alterations after irradiation at 10D or 10W

To further address the role of mitochondrial function and oxidative stress on irradiation-mediated responses, the enzymatic activities of the mitochondrial electron transport chain (ETC) complexes I-IV were evaluated in the hippocampus of 10D- and 10W-exposed mice at different time points after irradiation.

**Figure 5:**
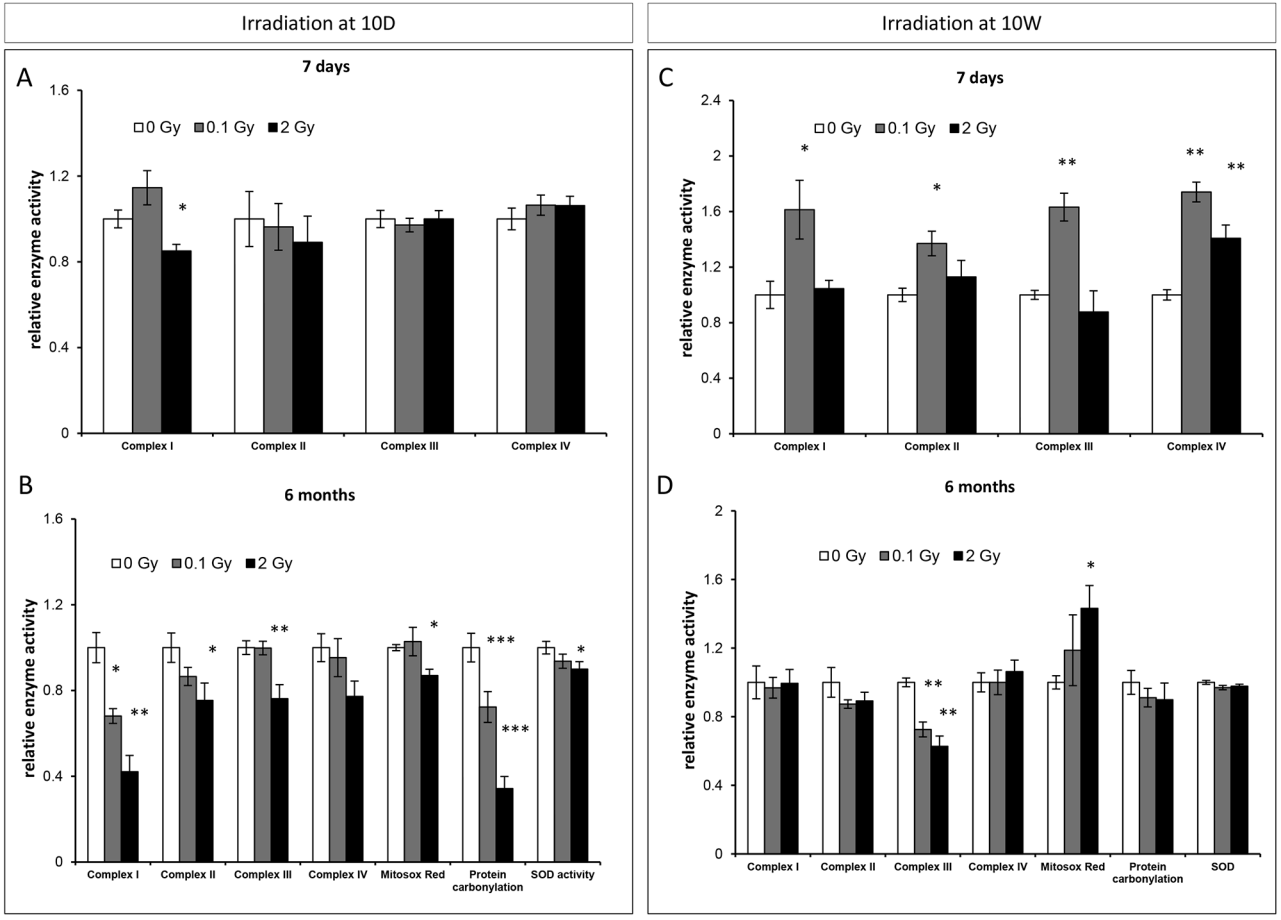
Radiation-induced mitochondrial damage in the hippocampus **A.** and **C.** Relative enzymatic activity of the mitochondrial electron transport chain (ETC) complexes I-IV in the hippocampus of 10D- or 10W-irradiated mice at 7 days, after irradiation. **B.** and **D.** Relative enzymatic activity of the mitochondrial ETC complexes I-IV, reactive oxygen species (ROS), carbonylated proteins and level of superoxide dismutase (SOD), in the hippocampus of 10D- or 10W-irradiated mice at 6 months postirradiation. Data are presented as mean ± SEM. *P < 0.05 or **P < 0.01 for comparison with controls. The number of animals used is n = 4–5.

In 10D-irradiated mice, at 7 days after 2 Gy exposure, mitochondrial alterations were manifested as a moderate, yet significant reduction in the activity of complex I (15%, Figure 5A). Six months after irradiation with 2 Gy, we observed significant downregulations of most of the components of the ETC essential to ATP production, including complexes I (58%), II (25%) and III (24%) (Figure 5B). Of note, even low-dose irradiation induced downregulation in complex I activity (32%; Figure 5B). The pattern of mitochondrial alterations in 10W-irradiated mice (Figure 5C, 5D) differed substantially, with a significantly increased enzyme activity for all complexes 1 week after low-dose irradiation (61% in complex I; 37% in complex II; 63% in complex III; 74% in complex IV), and an increase only in complex IV activity after irradiation with 2 Gy (41%) (Figure 5C). Also, radiation-induced late mitochondrial alterations markedly differed between 10D- and 10W-irradiated mice, being milder in the latter where only complex III was affected at both doses (Figure 5D). We next evaluated persistent oxidative stress and antioxidant defenses at 6 months post-irradiation in 10D- and 10W-irradiated mice by using the level of reactive oxygen species (ROS), carbonylated protein, superoxide dismutase (SOD) activity as biomarkers. Consistent with the marked downregulation of complex I, the major source of O_2_^−/−^ in the brain [35, 36] and complex III, we detected significant decreases of carbonylated protein, SOD activity and ROS levels in mice irradiated at 10D with 2 Gy (Figure 5B), suggestive of an adaptive program to avoid increase of radiation-induced oxidative stress. A significant decrease in carbonylated proteins was also detected at 0.1 Gy. Instead, in 10W-irradiated mice, in which only complex III was downregulated, the levels of carbonylated proteins and SOD were unchanged, while ROS levels were significantly increased (Figure 5D), suggesting that in mature hippocampus mitochondria are unable to modulate the cellular redox balance. Altogether, these data show that age at irradiation is an important determinant for the induction of adaptive processes in mitochondria. Low-dose irradiation caused an initial increase in the activities of the ETC enzymes in 10W- but not 10D-irradiated mice. Instead, irradiation at 10D but not at 10W, led to long-term reduction in the activities of the ETC enzymes.

### Persistent effects of irradiation at 10D or 10W on the hippocampal microvasculature

**Figure 6:**
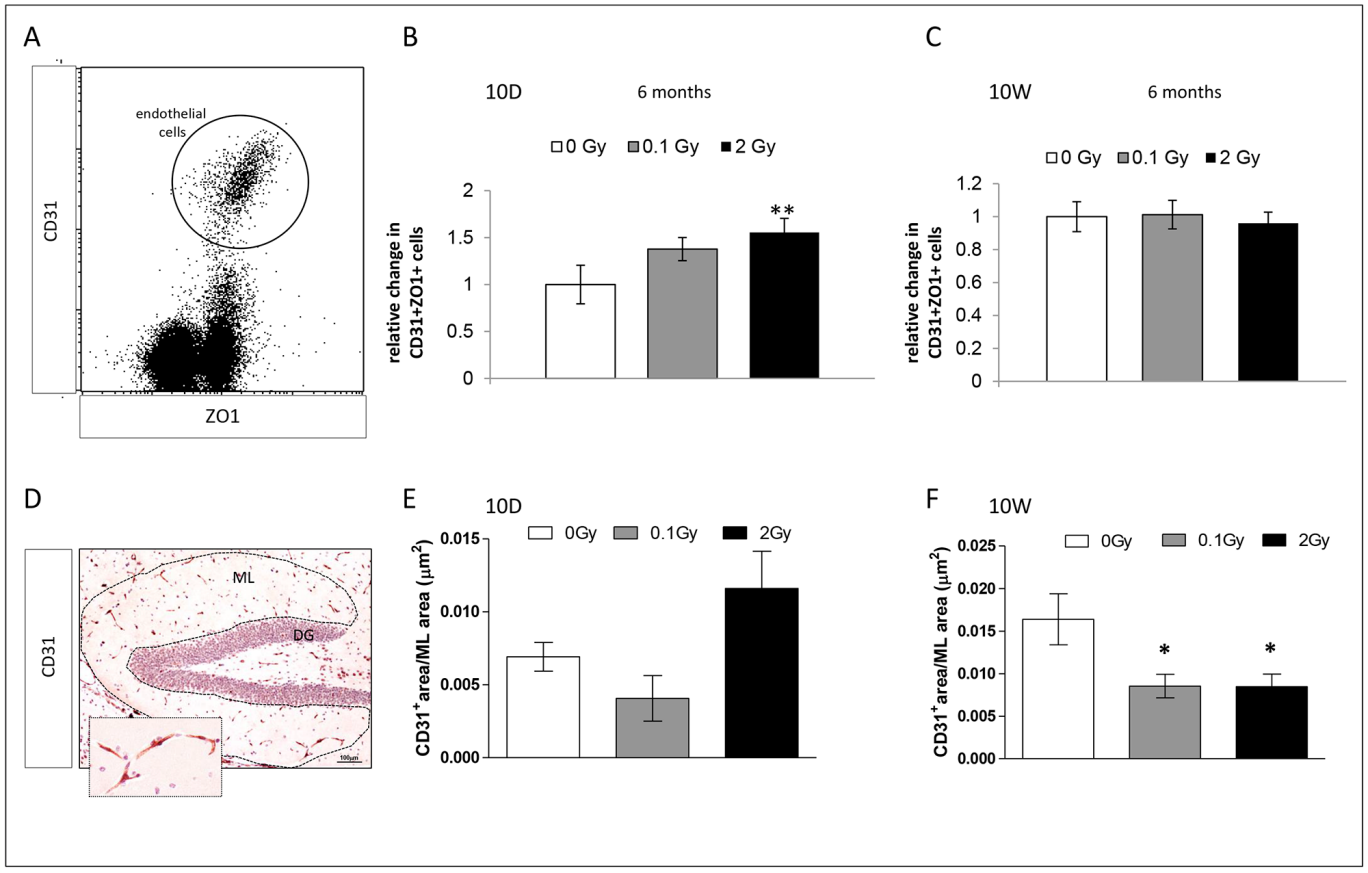
Endothelial changes in the hippocampus 6 months after irradiation at 10D and 10W **A.** Representative dot-plot of hippocampus single cell suspension CD31^+^/ZO^+^ cells are gated. **B.** Significant increase in the number of endothelial cells in 10D-irradiated mice. **C.** No significant changes in the number of endothelial cells in 10W-irradiated mice. **D.** Representative IHC image stained with antibody against CD31 for visualization of blood vessels. Image, 10x magnification, scale bar = 100 μm; high magnification 40x, scale bar 10 μm. **E.** and **F.** Quantification of microvessel total area in the ML (dashed line), showing no significant changes in 10D-irradiated mice (*P* = 0.08), as opposed to a significant decreases in 10W-irradiated mice (both at 0.1 and 2 Gy). Data are presented as mean ± SEM. *P < 0.05 or **P < 0.01 for comparison with controls. The number of animals used is n = 4–5.

To assess radiation-induced vascular changes, we evaluated the microvasculature of 10D- or 10W-exposed mice 6 months post-irradiation with different techniques (Figure 6). To quantify the microvessel level in the whole hippocampus by flow cytometry, endothelial cells were identified based on combined expression of a panendothelial marker (CD31) and a tight-junction marker (ZO1) (Figure 6A). In 10D-irradiated mice, a dose of 2 Gy induced a significant increase at endothelial cell level (CD31+/ZO1+; *P* = 0.002), indicating a chronic increase in microvasculature (Figure 6B). Instead, in 10W-exposed mice no chronic alterations were found (Figure 6C). Low-dose irradiation did not cause significant changes in hippocampus microvessel level at any of the investigated time points. Additionally, microvessels were histologically visualized with a CD31 staining (Figure 6D) and microvessels total area was quantified in the ML. Although not significant, a trend toward increased microvessels area was detected in 10D-irradiated mice (Figure 6E). Instead, compared to control mice, a significant reduction was found in 10W-irradiated mice both at low (*P* = 0.01) and high dose (*P* = 0.026; Figure 6F). Although cytometry and IHC data might appear discordant, different regions (whole hippocampus *vs* ML) have been evaluated. Altogether, these data point to opposite effects of irradiation at 10D and 10W on hippocampal microvasculature, with a tendency to increased density at 10D and decreased density at 10W.

### Cranial irradiation with low or moderate X-ray does not induce cognitive impairments

**Figure 7:**
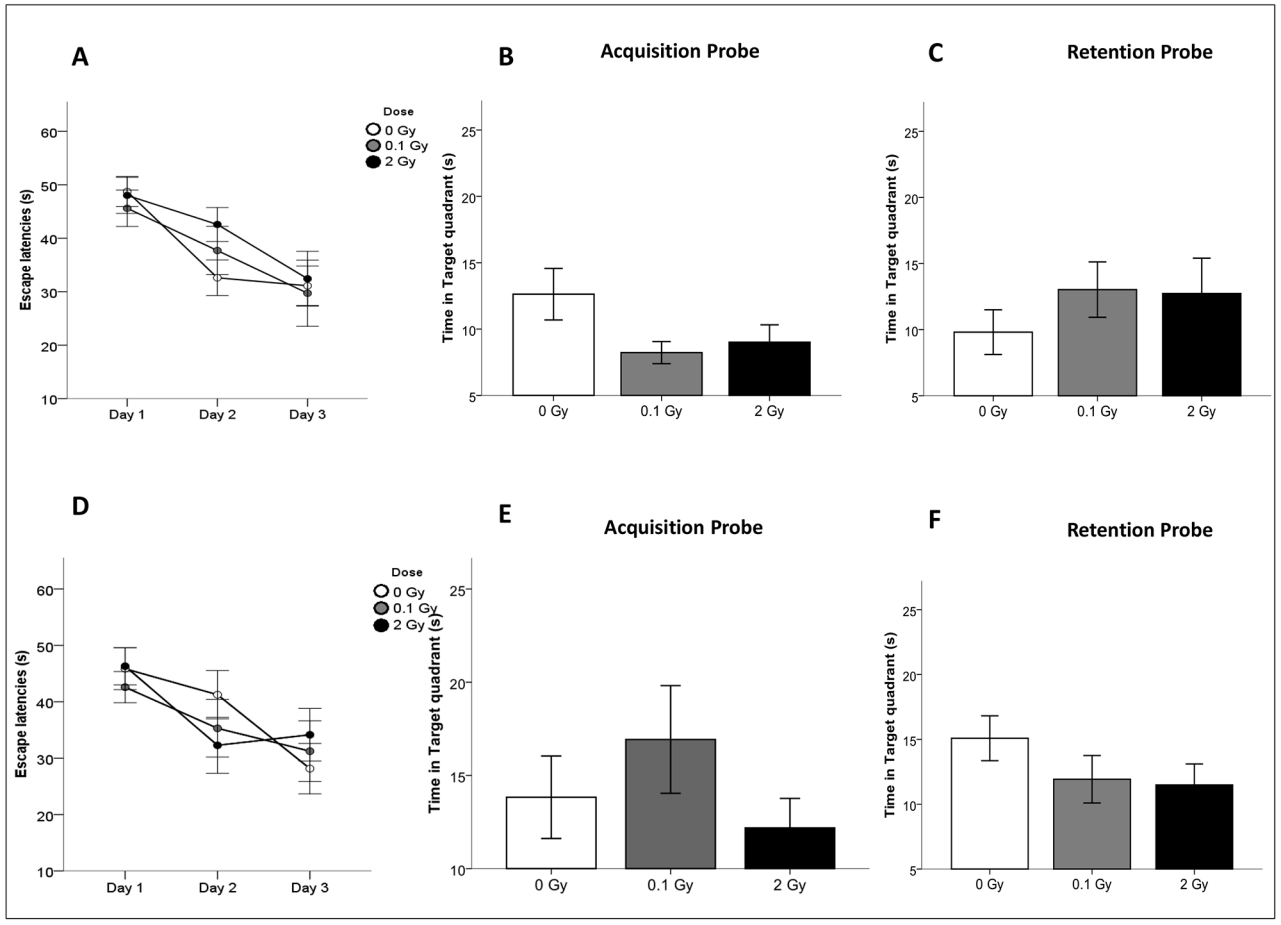
Cognitive performance after cranial irradiation at 10D or 10W with low or high X-ray doses **A.** and **D.** Escape latencies in the water maze test for acquisition days in 10D- and 10W-irradiated and control animals. **B.** and **E.** Time spent in target quadrant after the last training session (Acquisition Probe) and **C.** and **F.** 48 hours after the last training day (Retention Probe) of 10D- or 10W-irradiated and control mice at 6 months postirradiation. Data are presented as mean ± SEM. The number of animals used is n = 20.

To assess possible alterations in spatial learning and memory, we performed behavioral testing with the Morris water maze in both animal groups 6 months post irradiation (Figure 7). No significant difference was found in the success scores for both acquisition and retention of memory in the irradiated mice compared with controls (Figure 7B, 7C, 7E, 7F). To ensure that there was no difference between control and irradiated animals in regard to swimming or visual ability, the swim speed and the latency to reach the visible platform were also analyzed. No abnormalities were found (Figure 7A, 7D). Even though the learning process was slower for 10D-irradiated mice, all irradiated animals reached a comparable performance in the task until the end of the training period, indicating a similar level of learning for all groups. Similar experiments on NMRI mice whole-body irradiated (0.5–1 Gy) at 10D showed, instead, cognitive impairment, that was associated with chronic microglia activation [37]. Difference in the induction of cognitive effects between irradiated C57BL/6 and NMRI could either relate to differences of genetic background, irradiation modality (cranial vs whole-body), or behavioral tasks used (Morris water maze test *vs*. spontaneous exploratory behavior). Finally, the possibility of a transient cognitive impairment, fully recovered by 6 months post-irradiation, or of a later onset of the cognitive defects should also be considered.

## DISCUSSION

Cranial doses of ionizing radiation delivered to the brain of cancer patients can cause severe tissue damage, cognitive impairment and memory loss [38, 39]. Consequently, a great number of studies have dealt with the effects of radiation doses of therapeutic interest (≥ 45 Gy) in the CNS. The mechanisms of radiation responses at low doses (e.g., from diagnostic radiology) in the CNS, as in many other tissues, may completely differ from those at high doses, however, only a few studies have been conducted at doses ≤ 2 Gy. For these reasons, accurate risk evaluation of the late effects of cranial irradiation represents a crucial challenge, especially in children, who are at greater risk to develop learning disabilities as well as growth and psychomotor retardation [40, 41].

### Impaired neurogenesis and alteration of the synaptic plasticity in the irradiated hippocampus

Cranial irradiation of pediatric and adult patients elicits varying degrees of cognitive dysfunction through mechanisms such as inhibition of hippocampal neurogenesis [42], degradation of the neuronal structure and alteration of synaptic integrity and neuronal plasticity [43–45].

We show here that age at irradiation modifies the radiation response of the hippocampus. Irrespective of which cell type undergoes cell death, we found marked acute apoptosis in neonatal hippocampi 24h following irradiation with 2 Gy but not in 10W-irradiated mice, demonstrating a higher sensitivity of the neonatal compared to adult brain, consistent with hypersensitivity of proliferating cells to the killing effects of radiation. The neonatal DG undergoes rapid postnatal development, occurring through a burst of proliferation of granule cells precursors in the SGZ, with peak between postnatal days 7 and 14 [46]. Afterwards, proliferation progressively declines, and we show a sharp decrease (90%) in the relative number of cycling cells at 10W, thus explaining lack of cell killing at this time.

A more detailed analysis of cellular/molecular alterations induced by cranial X-ray irradiation during postnatal (10D) or adult neurogenesis (10W) using proliferation and lineage-specific maturation markers at several postirradiation times, shows that irradiation persistently altered cell dynamics within the DG. Lending further support to the higher radiosensitivity of young brains, 10D-, but not 10W-irradiated mice showed modification in the stem/proliferative cell compartment of the SGZ at 1 day postirradiation. Neurogenic effects showed a later occurrence in 10W-irradiated mice, becoming evident at 7 days after irradiation. Persistent depletion of cycling precursors, coupled with a deficit of the immature/mature granule neurons (Dcx/NeuN), was detected 6 months postirradiation in the SGZ of the DG of both 10D and 10W-irradiated mice. This is consistent with the notion that, as neurons are generated through extensive division of precursors, continued loss of neurons may deplete the precursor cell compartment. Noteworthy, at low dose (0.1 Gy) the long-term effects in the DG were milder, consisting in depletion of the proliferative compartment in 10D-irradiated mice and increase in the number of integrated mature neurons in the 10W group.

Proteomic data strongly suggested that the effects of irradiation during postnatal neurogenesis at 10D were more severe compared to irradiation at 10W, as shown by the larger number of deregulated proteins at 6 month postirradiation in 10D- compared to 10W-irradiated mice (74 *vs* 38). Among the 3 proteins showing common deregulation in 10D and 10W-irradiated mice, we found long-term alterations of synaptic plasticity-associated cytoskeletal remodeling pathways Rac1-Cofilin pathway, which is typical after neonatal exposure [37]. Here, Rac1 downregulation was also observed 10W-irradiated groups, suggesting that impairment of this pathway is a persistent molecular fingerprint of irradiation in the hippocampus independent from age at exposure and could therefore be valuable for clinical use as a biomarker.

Among proteins specifically deregulated in 10D-irradiated mice, we detected reduced levels of presynaptic SNAP-25 and increased postsynaptic proteins PSD-95. Impairment of synaptic functions may lead to neuropsychiatric disorders collectively referred to as synaptopathies, and reduced SNAP-25 expression is often found in brain areas of psychiatric patients [47]. Importantly, it has been recently shown *in vivo* that reduction of SNAP-25 expression in CA1 hippocampal region leads to less functional dendritic spines, as well as to a defective PSD-95 dynamics [48]. Alterations of synaptic plasticity mediated by PSD-95, accompanied by reductions in dendritic complexity and spine density, have been shown after proton or γ-rays exposure [43, 49]. In addition, we reported long-term increase of PSD-95 expression after neonatal irradiation, suggesting that alteration of synaptic proteins is a general pathogenic mechanism of brain irradiation, especially at young age. An important outcome of our study is the finding that neuronal populations of different maturation are impaired depending on age at irradiation, i.e., persistent impairment of immature neurons (Dcx^+^) and of terminally differentiated neurons (NeuN^+^) is observed in 10W- and 10D-irradiated mice, respectively. Notably, SNAP-25 mutant mice are characterized by a marked decrease (60%) in NeuN immunoreactivity, consistent with an “immature DG phenotype” [50], suggesting that SNAP-25 protein downregulation may lock new neurons in an immature state, accounting for both lack of deficit in the compartment of newly generated neurons (Dcx^+^) and impairment of terminally differentiated neurons (NeuN^+^), characteristic of 10D-irradiated mice. Moreover, “immature DG”, has recently been recognized as a feature of patients with psychiatric disorders such as schizophrenia and bipolar disorder [51].

### Neuroinflammation and microvasculature alteration in the hippocampus after irradiation

Microglia, that represent ∼10% of the total glial population in the CNS, are the macrophage equivalent of the CNS, where they serve as key mediators of neuroinflammation. High-dose irradiation frequently induces chronic microglia activation in the hippocampus [19, 52], which, through release of pro-inflammatory cytokines, inhibits proliferation of neural precursor cells and hippocampal neurogenesis [19, 52, 53]. Here, similar to what we observed after mouse prenatal exposure [54], we did not detect chronic microglia activation up to 6 months post-irradiation in both 10D- and 10W-irradiated mice, except for an initial pulse in Iba-1 expression at 1 day after cranial irradiation of 10D mice (2 Gy), excluding a causative role for chronic microglia activation in depression of hippocampal neurogenesis after cranial irradiation at low/moderate dose.

Once viewed as playing a mere supportive role, astrocytes are now recognized as a heterogeneous class of cells that perform diverse functions, including modulation of synaptic transmission and secretion of neurotrophic factors to promote neurogenesis [55, 56]. When activated in response to injury, astrocytes proliferate and upregulate GFAP protein [57]. Concordantly, we report an increase in the number of GFAP^+^ astrocytes in the H and ML during chronic phase (6 months) after cranial irradiation with 2 Gy at 10D, but not at 10W. Although limited information is available, results on GFAP activation after whole-body low-dose irradiation (≥ 0.1 Gy) at 10D, and cranial high-dose irradiation (≥ 20 Gy), have been reported by our and other groups [37, 58, 59]. While the functional significance of radiation-induced activation of astrocytes remains to be elucidated, they have been involved in the protection of endothelial cells from oxidative injury [60]. Our data provide support to this hypothesis by showing that GFAP activation correlates with reduced levels of ROS and increased microvessel density in the hippocampus after irradiation at 10D. Absence of GFAP activation in mice irradiated at 10W is consistent with early reports using adult mice, showing significant increases in astrocytes number only after doses > 30 Gy [58], and further stresses the importance of age-at-exposure in radiation-induced responses in the hippocampus.

### Mitochondrial dysfunctions and alterations of the redox system in the irradiated hippocampus

Another major pathophysiological mechanism by which ionizing radiation contribute to chronic alterations in the hippocampal functionality is the induction of mitochondrial dysfunctions [61–63]. Our network analysis of proteins chronically deregulated in 10D- and 10W-irradiated hippocampi demonstrates functional integration in pathways involved in “oxidative phosphorylation”, “mitochondrial dysfunction” and “superoxide radicals degradation”. Overall, analysis of mitochondrial ETC enzymes showed marked differences both in acute and chronic alterations induced in 10D- and 10W-exposed mice, suggesting a possible involvement of mitochondria in age-dependency. In particular, in the short-term all mitochondrial enzymes (Complexes I–IV) showed increased activity in mice exposed as adults, especially at 0.1 Gy. The sharp increase of nearly all complexes at 0.1 Gy was an unexpected and novel result, indicating an acute enhancement of mitochondrial functions after low-dose irradiation.

Little information exists on radiation-induced alterations in mitochondrial enzymes in the brain. Increased expression level of Complex I and Complex III proteins was reported in hippocampal neurons isolated from rat brain at embryonic day18 after *in vitro* irradiation with 0.2 Gy [64]. This increase was ascribed to mitochondrial fusion as part of an adaptive mechanism regulating neuronal survival. Despite the obvious difference in the experimental set-up, our data fit well with the adaptive hypothesis, as we detected a concomitant increase in the density of mature neurons in the DG of 10W mice 6 months after irradiation with 0.1 Gy, suggesting that early upregulation of mitochondrial metabolism might increase neuronal survival in the mature brain.

Interestingly, at 6 months postirradiation the relative susceptibility of 10D and 10W irradiated mice was fully reversed and most mitochondrial ETC complexes (i.e., I, II and III) were downregulated in 10D-irradiated mice, compared to downregulation of complex III only in 10W-irradiated mice. Importantly, the alterations in ETC complexes and SOD1 activity detected by functional analysis were largely confirmed by proteomic data, supporting age-related differences in the mechanisms of radiation-induced response in hippocampal mitochondria. Modulation of mitochondrial complexes activity, leading to functional reduction in mitochondrial respiratory capacity, was also observed after neonatal irradiation (0.1–2 Gy) of C57BL/6 mice [65, 66]. Although we cannot mechanistically explain why exclusive for neonatal irradiation, we could speculate that a chronic reduction of mitochondrial activity might represent a general mechanism to prevent oxidative stress preserving basic cellular functions during hippocampus development. Indeed, our results clearly indicate age-related differences in maintenance of redox balance in the hippocampus after stress, shown by reduction in ROS levels associated with the chronic Complex I decrease in 10D-irradiated mice. However, if preventing oxidative damage through mitochondrial downregulation, as in Down syndrome, might be an adaptive response for avoiding excessive ROS generation, on the other hand, impairing cellular functions with high-energy demands, such as synaptic transmission, might predispose to conditions associated with altered energy metabolism, such as Alzheimer disease, autistic spectrum disorders and Leigh disease [67, 68]. Important age-related differences in radiation-induced stress response are also suggested by persistent upregulation of a higher numbers of heat-shock proteins (Hsps) following irradiation at 10D (Hsp8, Hsp60, Hsp70 and Hsp90), compared to 10W (Hsp60). These proteins are a broad family of key protective factors controlling protein homeostasis that are upregulated in response to stress [69] and may help counteract the increase of damage in the brain and subsequent neurodegeneration process.

Overall, our study gives a broad picture of hippocampal response to cranial irradiation of neonatal or adult mice, highlighting substantial age-related differences. Developing hippocampus mounts a persistent antioxidant defense in response to irradiation, consisting in induction of a prominent stress response (Hsps) and downregulation of nearly all mitochondrial complexes, with consequent decrease of oxidative stress (ROS and protein carbonylation). Mechanistically, this can be interpreted as a protective strategy to avoid neurodegeneration. Nevertheless, as mitochondria are important regulators of fundamental processes in neuroplasticity, including neural differentiation, neurite outgrowth, neurotransmitter release and dendritic remodeling [70], disturbances in mitochondrial functions might lead to alterations in pre- and post-synaptic apparatuses, and these defects of neuronal connectivity might, in turn, result in depletion of granule neurons. Even though these alterations were insufficient to cause overt cognitive impairment of 10D- and 10W-irradiated mice at 6 months postirradiation, a trend toward a slower learning process was observed in 10D-irradiated mice. In humans radiation-induced cognitive decay often shows a late occurrence, developing many years after exposures to high doses of therapeutic interest, leaving open the possibility of a later onset for development of overt cognitive deficits in mice irradiated with low/moderate doses.

## CONCLUSIONS

We assessed global stress responses in the hippocampus of mice irradiated at different ages (10D *vs* 10W) with different doses of X-rays (0.1 *vs* 2 Gy). Our *in vivo* study provides evidence that age at irradiation influences radiation response, particularly when irradiation occurs during development of the hippocampus (10D), as indicated by increased apoptosis, and by long-term alterations in mitochondrial homeostasis, neurogenesis and expression of proteins involved in synaptic plasticity. We propose that the different effects of irradiation on mitochondrial homeostasis and redox balance in developing and mature hippocampus might be central to the age-dependency of radiation-induced responses. We also show that a dynamic interaction of multiple cell types, such as neural precursor cells, neurons, astrocytes and endothelial cells, may be involved in the detrimental consequences of irradiation in the hippocampus. Importantly, some of the cellular/molecular effects are also induced by a low radiation dose (0.1 Gy) - in the range of medical diagnostic exposure - suggesting the need for further investigations on this issue. Whether the changes elicited by low/moderate radiation doses are potentially detrimental by synergizing with other genetic/environmental factors remains to be addressed. Our study sheds light on key cellular/molecular alterations involved in brain radiation sensitivity, as well as their dependence on age at irradiation and radiation dose, and its results may help ameliorate radiation therapy by minimizing radiation-associated health risks.

## MATERIALS AND METHODS

### Animal irradiation

Female and male C57BL/6 mice were purchased from Janvier labs (Le Genest-Saint-Isle, France) and bred at the animal facility of the National Research Directorate for Radiobiology and Radiohygiene, Budapest, Hungary. The experimental design and the time line of the study are shown in Figure 1 E. 10D or 10W old C57Bl/6 mice were cranially exposed to low (0.1 Gy) or moderate (2 Gy) doses of X rays. During the experiments animals were kept in individual cages (maximum 6 animals/cage) and were provided with standard food and water *ad libitum*. At the end of the experiments, animals were sacrificed by cervical dislocation.

All animal studies were conducted according to the 1998 XXVIII Hungarian law about animal protection and welfare. Animal studies were approved and permission was issued by Budapest and Pest County Administration Office Food Chain Safety and Animal Health Board (permit number: 22.1/2703/3/2011).

### Tissue collection and immunohistochemistry analysis

Brains from unirradiated (n = 4–5) and irradiated (n = 4–5) mice were collected at different time points (Figure 1E), fixed in 10% buffered formalin and embedded in paraffin wax according to standard techniques.

To analyze adult neurogenesis, the brain of each mouse was cut sagittaly to the midline and sections were collected starting at 500 μm from the midline. To standardize the counting area, cell quantification was performed on two non-overlapping serially collected sections per mouse, one from each hemisphere, representing the rostral/mid-hippocampus.

For staining with Caspase-3, GFAP, PCNA, Ki67, Sox2, Dcx, NeuN, CD31 and Iba1 antibodies, 4 μm-thick brain sagittal sections were dewaxed, rehydrated and heated in citrate buffer. Quenching of endogenous peroxidase was performed with 3% H_2_O_2_ in distilled water. Brain sections were incubated with primary antibody dilutions as indicated by the manufacturer: Caspase-3 (9664, Cell Signaling Technology, Danvers, MA, USA), GFAP (Z0334, Dako, Carpinteria, CA, USA), Sox2 (ab97959, abcam, Cambridge, UK), Dcx (ab18723, abcam), Iba1 (019-19741, Wako Pure Chemical Industries, Osaka, Japan), PCNA (NA03 mAB-1, Calbiochem, Germany), Ki67 (ab15580, abcam) and NeuN (MAB377, Millipore, Germany), CD31 (ab283664, abcam).

Immunohistochemical analysis of polyclonal antibodies against Sox2, Dcx and CD31 complexes was carried out using a rabbit biotin-conjugated secondary antibody; after incubation with avidin-biotin immunoperoxidase staining, the antibody-antigen complexes were visualized by Vector NovaRED Substrate Kit (Vector Laboratories, Inc., Burlingame, CA) according to manufacturer's instructions.

Antibody–antigen complexes of polyclonal GFAP or caspase-3 were visualized using horseradish peroxidase-conjugated secondary antibody and the DAB chromogen system (Dako). Immunohistochemical analysis of monoclonal antibodies against PCNA and NeuN was performed using the HistoMouse MAX Kit (Invitrogen Corporation, Camarillo, CA) according to manufacturer's instructions.

Cell quantification was performed on collected sections. For quantification of radial glial cells (RGL), microglia and endothelial cells, GFAP/Iba1/CD31-stained sections were imaged with HistoFAXS at 10x magnification. The subgranular zone (SGZ) area of the DG, H and the ML of hippocampus were analyzed by HistoQuest software for automatic color separation and quantification. The number of GFAP/Iba1/CD31-positive cells was expressed as the fraction of the labeled area out of the total SGZ, H or ML area. For PCNA, Sox2, and Dcx quantification, the number of positive cells in the SGZ was expressed per DG area (μm^2^), measured by the imaging software NIS-Elements BR4.00.05. Immunohistochemical staining for NeuN was performed to assess the neuronal density in the granule cell layer of the DG. Counting was carried out in a rectangular field of 2000 μm^2^ in the supra- and infrapyramidal blade and in the crest area of the DG. The number of positive cells in each of the areas was carried out from the mean from 4/5 biological replicates. All the images were analyzed using identical software settings.

### Tissue collection and protein isolation for proteomic analysis

Animals were killed 6 months post-irradiation via cervical dislocation (10D and 10W sham-irradiated and 2 Gy irradiated mice). Brains were excised and transferred to ice-cold phosphate buffered saline (PBS), rinsed carefully, and dissected under stereomicroscopic inspection under cold conditions (ice-cold PBS). Hippocampi from each hemisphere were separately sampled, gently rinsed in ice-cold PBS and snap-frozen in liquid nitrogen. Samples were shipped in dry ice to Helmholtz Zentrum München, Munich, Germany, and were stored at −80°C until isolation of proteins. Hippocampi from 4 biological replicates per dose group and time point were manually homogenized with 6 M guanidine hydrochloride (SERVA Electrophoresis GmbH, Germany) on ice using a manual plastic mortar. Subsequently, the homogenates were briefly vortexed, sonicated, and cleared by centrifugation (20,000g, 1 h, 4°C). Supernatants containing total protein content were collect and stored at −20°C before further analysis. Protein concentration was determined by Bradford assay (Thermo Fisher) following manufacturer's instructions.

### Mass spectrometry-based proteome analysis

The isotope coded protein label (ICPL quadruplex) method was used to quantify proteome alterations as recently described [37]. Briefly, the protein lysates (20 μg in 20 μl of 6 M guanidine hydrochloride from each biological replicate) were reduced, alkylated and labelled with the four different ICPL-tags as follows: control of 10D with ICPL-4, control of 10W with ICPL-0, 2 Gy sample of 10D with ICPL-10 and 2 Gy sample of 10W with ICPL-6. The four labelled samples were combined and overnight precipitated with 80% acetone at −20°C to purify the labelled protein content. Four biological replicates in each dose group were used. Protein precipitates were separated by 12% SDS-polyacrylamide gel electrophoresis followed by Coomassie Blue staining. Gel lanes were cut into five equal slices, destained, and trypsinized overnight as described previously [71]. Peptides were extracted and acidified with 1% formic acid followed by analysis via mass spectrometry. LC-MS/MS analysis was performed as described previously on a LTQ-Orbitrap XL (Thermo Fisher) [72]. Briefly, the pre-fractionated samples were automatically injected and loaded onto the trap column (Acclaim PepMap100, C18, 5 Qm, 100 Å pore size, 300 Qm ID × 5 mm Q-Precolumn - No 160454; Thermo Scientific). After 5 min, the peptides were eluted and passed to the analytical column (Acclaim PepMap100, C18, 3 Qm, 100 Å pore size, 75 Qm ID × 15 cm, nanoViper - No 164568; Thermo Scientific) by reversed phase chromatography which was operated on a nano-HPLC (Ultimate 3000, Dionex). A nonlinear 170 min gradient was used for elution with a mobile phase of 35% acetonitrile in 0.1% formic acid in water (A) and 0.1% formic acid in 98% acetonitrile (B) at a flow rate of 300 nl/min. The gradient settings were: 5–140 min: 14.5-90% A, 140–145 min: 90% A - 95% B, 145–150 min: 95% B followed by equilibration for 15 min to starting conditions. The 10 most abundant peptide ions were selected from the MS pre-scan for fragmentation in the linear ion trap if they exceeded an intensity of at least 200 counts and were at least doubly charged. During fragment analysis via collision-induced fragmentation (collision energy: 35 V), a high-resolution (60.000 full-width half maximum) MS spectrum was acquired in the Orbitrap with a mass range of 300 to 1500 Da. Target peptides were dynamically excluded for 60 seconds if already selected for MS/MS.

MS-MS spectra were searched against the ENSEMBL mouse database (Version: 2.4, 56416 sequences) via MASCOT search engine (version 2.3.02; Matrix Science). A mass tolerance of 10 ppm for peptide precursors and 0.6 Da for MS-MS peptide fragments was applied allowing not more than one missed cleavage. Fixed modifications included carbamidomethylation of cysteine and ICPL-0, ICPL-4, ICPL-6 and ICPL-10 for lysine. Proteins were identified and quantified based on the ICPL pairs (ICPL10/ICPL4 for day10 samples and ICPL6/ICPL0 for week10 samples 6 month post-irradiation) using the Proteome Discoverer software (Version 1.3 – Thermo Fisher). To ensure that only high-confident identified peptides were used for protein quantification, the MASCOT percolator algorithm was applied (q value filter of 0.01). Subsequently, these peptides were filtered against a Decoy database resulting into a false discovery rate (FDR) of each LC-MS-run; the significance threshold was set to 0.01 to ensure that only highly confident peptide identifications were used for protein quantification. Proteins from each LC-MS-run were normalised against the median of all quantifiable proteins. Proteins were considered to be significantly deregulated if they fulfilled the following criteria: (i) identification by at least two unique peptides in n-1 mass-spectrometry runs (n: number of biological replicates), (ii) quantification with an ICPL-variability of ≤ 30% and (iii) a fold-change of ≥ 1.3 or ≤-1.3. A threshold for the fold-change of ±1.3 was used, based on our average experimental technical variance of the multiple analyses of hippocampal technical replicates [37].

The raw-files of the obtained MS-MS spectra are deposited at http://storedb.org/project_details.php?projectid=48 with the ProjectID 48.

### Bioinformatics analysis of proteomic data

Deregulated proteins were categorized into protein classes using PANTHER (Protein Analysis Through Evolutionary Relationships) classification system software (http://www.pantherdb.org) and the general annotation from UniProt (http://uniprot.org). We used the STRING software (http://string-db.org/) to evaluate the protein-protein interactions of the deregulated proteins. The analyses of affected signaling pathways from all deregulated proteins were performed with the INGENUITY Pathway Analysis (http://www.ingenuity.com) software. Deregulated proteins with their protein accession number and fold-changes were imported into the IPA core analysis, followed by a hierarchical comparison analysis. The IPA comparison analysis takes into account the signaling pathway rank according to the calculated *P*-value and reports it hierarchically. The software generates significance values (*P*-values) between each biological or molecular event and the imported proteins based on the Fischer's exact test (*P* ≤ 0.05). We used only the database information of experimental and predictive origin regarding CNS to be confident about the potential affected signaling pathways in the hippocampus.

### Immunoblotting analysis

Proteins separated by 4-12% SDS-PAGE were transferred to nitrocellulose membranes (GE Healthcare) using BIO-RAD Criterion™ Blotter system at 100 V for 2 h. The membranes were blocked using 8% milk in PBS, pH 7.4, for 2 h at room temperature, washed three times in 10 mM Tris-HCl, pH 7.4, 150 mM NaCl for 5 min and incubated overnight at 4°C with primary antibodies using dilutions recommended by the manufacturer. Immunoblot analysis of hippocampus protein lysate was performed using anti- postsynaptic protein PSD-95 (Dlg4) (ABIN1742271, Atlanta, GA, USA), anti-Rac1 (ab33186, abcam), anti-Hspd1 (ab46798, abcam), anti-Sod1 (sc-11407, Santa Cruz Biotechnology, Dallas, Texas USA), and anti-tubulin alpha (GTX72360, Biocompare, San Francisco, CA, USA). After washing three times, the blots were incubated with alkaline phosphatase-conjugated anti-mouse, or anti-rabbit secondary antibody (Santa Cruz Biotechnology) for 2 h at room temperature and developed using 1-step™ NBT/BCIP method (ThermoFisher) following standard procedures. The level of alpha-tubulin was used for normalization. Quantification of digitised images of immunoblot bands was done using ImageJ (http://rsbweb.nih.gov/ij/).

### Dissection of mouse hippocampus and preparation of single cell suspension

After cervical dislocation the hippocampus was dissected from whole brains. Hippocampal tissue was finely chopped, followed by enzymatic digestion, which was done with the Miltenyi Neural Dissociation Kit (Miltenyi Biotec GmbH, Bergisch Gladbach, Germany) according to the manufacturer's instructions. To separate the cellular fraction from lipid/myelin debris the resulting one cell suspension was further purified with OptiPrep (Alere Technologies AS, Rodelokka, Norway) gradient solutions. Single cell suspensions in HBSS were carefully layered on top of the Optiprep gradient. After centrifugation the top layer constituted the lipid/myelin debris, and underneath were the various cell fractions in distinct layers.

### Phenotypical analysis of brain endothelial cells

CD31ZO-1 double positive hippocampus microvascular endothelial cells were identified using an anti-CD31 antibody (eBioscience, San Diego, USA) and an anti-ZO1 antibody (Santa Cruz Biotechnology). Cell surface staining of freshly isolated brain single cell suspensions was performed with the fluorescently labelled antibodies at 4°C for 30 minutes. All flow cytometry measurements were carried out using a FACSCalibur flow cytometer (Becton Dickinson, San Jose, CA, USA). Analysis was performed using CellQuestTM software, version 3.1 (Becton Dickinson).

### Isolation of mitochondria enriched fraction from mouse brain

The dissected mouse hippocampus region was homogenized with a Dounce homogenizer in mitochondrial isolation buffer (0.32 M sucrose, 1 mM K-EDTA, 10 mM Tris-HCl, pH: 7.4) on ice. Brain homogenates were subjected to two successive centrifugation steps (370 g at 4°C for 10 minutes). The removed supernatants were centrifuged with 12000 g at 4°C for 10 minutes, the resulting pellets were suspended in mitochondrial isolation buffer and centrifugation was repeated. The concentration of enriched mitochondria fraction was determined by Bradford method (Thermo Fisher Scientific Inc. Waltham, MA, USA). The final pellets, containing the mitochondria, were suspended in 100 μl of mitochondrial isolation buffer and stored at −80°C until use.

### Quantification of enzyme activities of Complex I-IV of the mitochondrial electron transport chain (ETC)

Complex I (NADH-ubiquinone reductase) and II (Succinate dehydrogenase) activity was determined by measuring the reduction of 2,6-Dichlorophenolindophenol (DCPIP) at 600 nm wavelength. To determine complex I activity, 50 μg/ml mitochondrial extract was incubated with a solution containing 25 mM K-phosphate (pH 7.4), 3.5 g/l BSA, 60 μM DCPIP, 70 μM decylubiquinon, 1 μM Antimycin-A, 2 μM KCN at 37°C for 3 min. The reaction was initiated with 0.2 mM NADH, and the enzyme activity was quantified by measuring reaction kinetics both in the presence and absence of 0.2 mM Rotenone (specific inhibitor of Complex I) for 2 min. To determine complex II activity, 50 μg/ml mitochondrial extract was incubated with a solution containing 80 mM K-phosphate buffer (pH 7.4), 1 g/l BSA, 2 mM EDTA, 0.2 mM ATP, 80 μM DCPIP, 50 μM Q1 Coenzyme, 1 μM Antimycin-A, 3 μM Rotenone, 0.3 mM KCN at 37°C for 8 min. The reaction was initiated with 10 mM Succinate, and enzyme activity was quantified by measuring reaction kinetics both in the presence and absence of 1 μM 2-Thenoyltrifluoreacetone (TTFA, specific inhibitor of Complex II) for 3 min. Complex III (Cytochrome c oxido-reductase) activity was determined by measuring the reduction of cytochrome c at 550 nm. The assay was performed in a buffer containing 25 mM K-phosphate (pH 7.4), 1 mM EDTA, 1 mM KCN, 32 μM cytochrome-c and 0.6 mM dodecyl maltoside, using 15 μg/ml mitochondrial extract. The reaction was initiated with freshly made 35 μM decylubiquinol solution. The reaction was quantified by measuring reaction kinetics both in the presence and absence of 2 μM Antimycin-A (specific inhibitor of Complex III) for 1 min at 30°. The decylubiquinone was reduced by adding K-borohydrate and 1 M HCl. Complex IV (Cytochrome c oxidase) activity was determined by measuring the oxidation of cytochrome-c at 550 nm. The 0.22 mM cytochrome c was reduced by adding 0.5 mM Dithiothreitol (DTT). The reaction was performed in a buffer containing 10 mM Tris-HCl (pH 7.0), 120 mM KCl, 25 mM sucrose, 11 μM reduced cytochrome-c and 2 μg/ml mitochondrial extract. The activity was quantified by measuring reaction kinetics for 1 min at 30°C. All measurements were carried out using BioTek Synergy HT plate reader (Biotek Instruments, Winooski, Vermont, USA). All reagents used for quantification of enzyme activities, unless specified otherwise, were purchased from Sigma (Sigma-Aldrich Inc. St. Louis, MO, USA).

### Evaluation of mitochondrial ROS levels by flow cytometry using the Mitosox Red assay

For the quantification of mitochondrial ROS levels, hippocampus single cell suspension was used, prepared as described above. Cells were stained with the Mitosox Red dye (Life Technologies, Carlsbd, CA, USA) according to the manufacturer's instructions and analysed by flow cytometry as described above.

### Evaluation of mitochondrial ROS levels by quantifying mitochondrial protein carbonylation levels and mitochondrial antioxidant capacity by measuring mitochondrial SOD levels

Protein carbonylation was measured by OxiSelectTM Protein Carbonyl ELISA Kit according to manufacturer recommendations (Cell Biolabs, San Diego, USA), using 10 μg/ml mitochondria enriched fraction. Oxidative agents can cause covalent modification of protein. Protein carbonyl group is relatively stable derivatives of oxidative stress, which can interact with 2,4-dinitrophenylhydrazine (DNPH), and evolve stable dinitrophenyl (DNP) hydrazone product. This product can be detected by using anti-DNP antibody in an enzyme immunoassay system. SOD activity level was determined by SOD determination kit (Sigma-Aldrich) according to the manufacturer's recommendations, using 500 μg/ml mitochondria enriched fraction. Briefly, Sigma SOD determination kit uses indirect method for determine SOD activity by using WST-1 (2-(4-Iodophenyl)- 3-(4-nitrophenyl)-5-(2,4-disulfophenyl)- 2Htetrazolium, monosodium salt). Xantine oxidase (XO) converts xanthine and O_2_ to uric acid and H_2_O_2_. Superoxide anion converts the tetrazolium salt of WST-1 into formazan dye. Adding of SOD to the reaction reduces the level of superoxide, and inhibits the conversation of formazan dye from WST-1. SOD activity was quantified by measuring the absorbance at wavelength of 450 nm 20 min after the reaction was started. The SOD activity was calculated from inhibition rate.

### Behavioral testing Water maze test (WM)

To evaluate spatial learning and memory, 10D- or 10W-irradiated C57BL/6 mice were subjected to water maze (WM) test (Morris) at 6 months postirradiation. The WM consisted of a circular tank (diameter 1 m; height 60 cm), divided into four quadrants. An escape platform (diameter 10 cm) was located 1 cm below the surface of the water in the target quadrant. Animals performed 5 trials per day for 3 consecutive days. During each trial, mice were allowed 60 s to find the hidden platform and to remain on it for 30 s. If the animal failed to find the platform within this period, it was placed on it by the experimenter. The order of the three starting positions was randomized throughout the day for each mouse. Extra-maze clues were located around the pool to provide a spatial configuration of the task. To avoid proximal cues and prevent egocentrical learning, an internal mobile wall was added to the maze, being the wall randomly moved between trials. This seems to increase Morris water maze sensitivity [73, 74]. At the end of the third acquisition day, the task was assessed by a probe trial, which consisted of a 60 s free swim without the escape platform (Acquisition Probe). An additional probe trial to evaluate the spatial memory of animals (Retention Probe) was performed 48 h after the last training day. Animal performance was recorded using a video camera placed above the maze. Data were analysed by the video tracking program Ethovision XT© (Noldus Information Technologies, Wageningen, The Netherlands). Latency to escape the platform during the training sessions was measured. During the probe trial, total time spent in the target quadrant, as well as the time spent in other quadrants were also measured in order to compare the time spent searching in the target quadrant between groups.

### Statistical analysis

Statistical analyses were performed using GraphPad Prism 5.0 software. Statistical significance was determined using a two-tailed student's t-test for comparison between pairs of means. *P*-values < 0.05 were considered to be statistically significant and marked with * if *P*<0.05, ** if *P*<0.01 or ***if *P*<0.001. Error bars represent standard errors (SE).

Cognitive data are presented as the mean ± standard error of the mean (SEM). Homogeneity of variances was analysed using the Levene's test. If variances were homogeneous, ANOVA was used followed by the Tukey post hoc test to evaluate all dose groups simultaneously. If the variances were not homogeneous, the Kruskal–Wallis test was then used. Differences between groups were analysed using the Mann–Whitney U-test. Moreover, the paired t-test was used to compare the two different points of time tested. The ANOVA test for repeated measures and post hoc analyses adjusted by Bonferroni's correction were used to analyse the progression of parameters recorded by the Ethovision XT© software. The level of statistical significance for all tests was established at *P* < 0.05. All data were analysed by means of the statistical package SPSS© v.21 (SPSS Sciences, Chicago, USA).

## SUPPLEMENTARY FIGURES AND TABLES






