# Supplementary material for: Age-related effects of X-ray irradiation on mouse hippocampus
Source: Oncotarget. 2016 Apr 4;7(19):28040–58. doi: 10.18632/oncotarget.8575 (PMC5053708; doi:10.18632/oncotarget.8575)
Supplement: Supplementary file 1 [file oncotarget-07-28040-s001.pdf]

SUPPLEMENTARY FIGURES AND TABLES

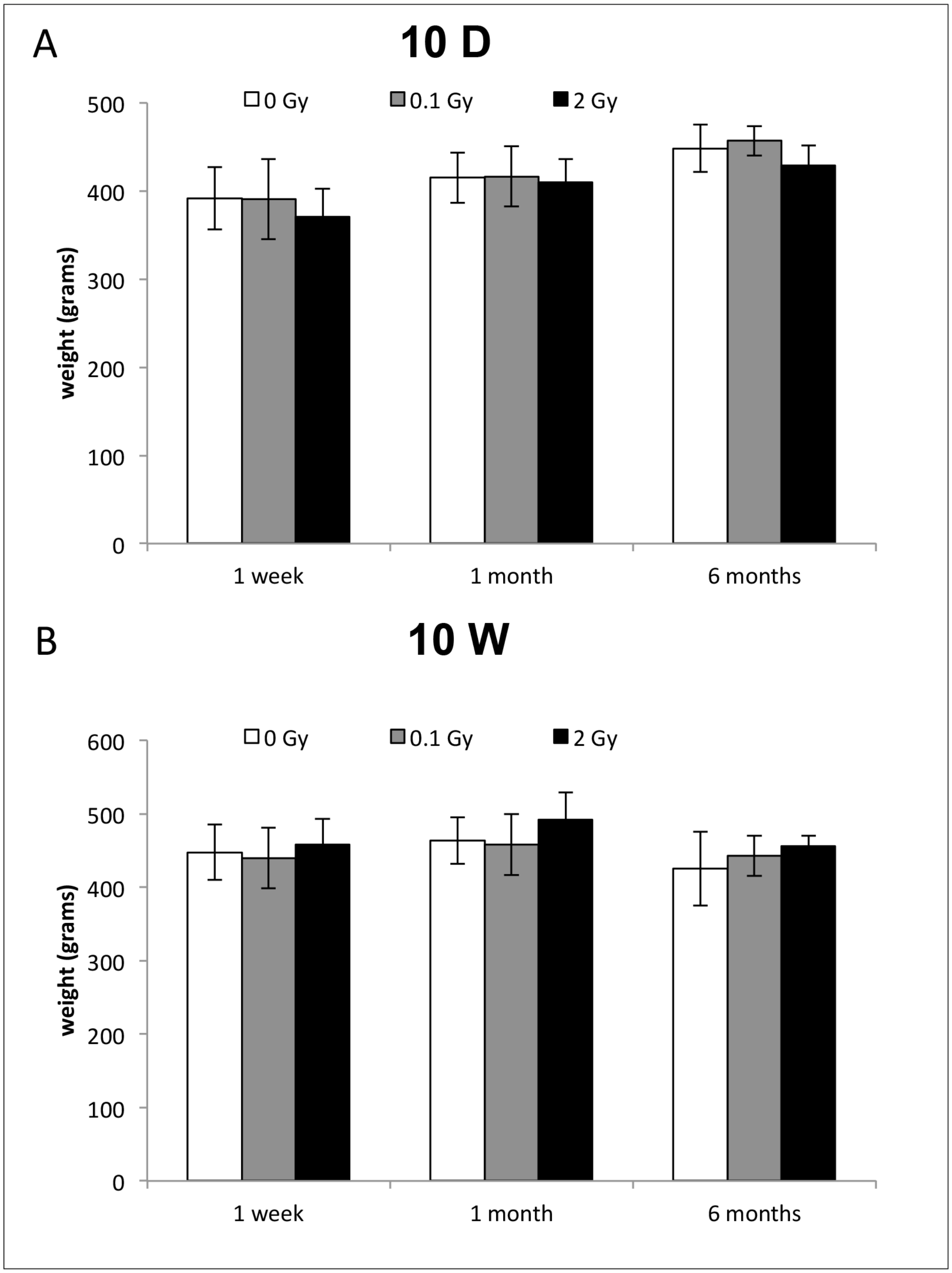

Supplementary Figure S1: Lack of differences in brain weight between control and irradiated mice. A. Brain weight at 1 week, 1 month or 6 months after 10D-irradiation with 0.1 or 2 Gy of X-rays and in age-matching control mice. **B.** Brain weight at 1 week, 1 month or 6 months after 10W-irradiation with 0.1 or 2 Gy of X-rays and in age-matching control mice.

Supplementary Table S1: Proteomics experiments via repetitive measurement of technical replicates (S1 10D). List of quantifiable hippocampal proteins 6 months after cranial irradiation with 2 Gy of X rays at 10D using proteome analysis.

See Supplementary File 1

Supplementary Table S2: Proteomics experiments via repetitive measurement of technical replicates (S2 10W). List of quantifiable hippocampal proteins 6 months after cranial irradiation with 2 Gy of X rays at 10W using proteome analysis.

See Supplementary File 2
